# Effect of active teaching strategies on interprofessional clinical judgment: a quasi-experiment

**DOI:** 10.1590/0034-7167-2024-0148

**Published:** 2025-03-14

**Authors:** Susi Cristalino Pereira, Mikael Lucas Anjo Pires, Paulo Percio Mota Magro, Tayse Tâmara da Paixão Duarte, Marcia Cristina da Silva Magro

**Affiliations:** I Hospital Universitário de Brasília. Brasília, Distrito Federal, Brazil; II Universidade de Brasília. Brasília, Distrito Federal, Brazil; II Instituto Federal de Brasília. Brasília, Distrito Federal, Brazil

**Keywords:** Clinical Reasoning, Interprofessional Education, Teaching, Intensive Care Units, Heart Arrest., Razonamiento Clínico, Educación Interprofesional, Enseñanza, Unidades de Cuidados Intensivos, Paro Cardíaco.

## Abstract

**Objectives:**

to assess the effect of active teaching strategies on clinical judgment for cardiopulmonary arrest care of patients with COVID-19 in in-hospital settings by an interprofessional team.

**Methods:**

quasi-experimental study without a comparison group. A total of 85 professionals were selected by non-probabilistic sampling. The educational intervention consisted of a class combined with skills training. The significance level was 5%.

**Results:**

most professionals were categorized as “proficiency” on a clinical judgment scale (52.9%). The “exemplary” category accounted for 31.8% of the total. Only 2.4% of participants were categorized as “beginning” and 12.9% were “developing” post-intervention.

**Conclusions:**

an active strategy based on a dialogued lecture combined with skills training had a positive impact on clinical judgment improvement. Thus, participatory educational actions, based on an active teaching method, developed, in most nurses, the “proficiency” and “exemplary” levels, while, in the medical and physiotherapy team, the “exemplary” level predominated.

## RESUMEN

**Objetivos:**

evaluar el efecto de estrategias de enseñanza activa sobre el juicio clínico para el tratamiento de la parada cardiopulmonar en pacientes con COVID-19 en el ámbito hospitalario por parte de un equipo interprofesional.

**Métodos:**

estudio cuasiexperimental, sin grupo de comparación. Se seleccionaron 85 profesionales mediante muestreo no probabilístico. La intervención educativa fue una clase combinada con entrenamiento de habilidades. El nivel de significancia fue del 5%.

**Resultados:**

la mayoría de los profesionales fueron categorizados como “competentes” en una escala de juicio clínico (52,9%). La categoría “ejemplar” representó el 31,8% del total. Sólo el 2,4% de los participantes fueron categorizados como “principiantes” y el 12,9%, “en desarrollo” después de la intervención.

**Conclusiones:**

la estrategia activa basada en la conferencia dialogada combinada con el entrenamiento de habilidades tuvo un impacto positivo en la mejora del juicio clínico. Así, las acciones educativas participativas, basadas en un método de enseñanza activo, desarrollaron, en la mayoría de los enfermeros, los niveles “competencia” y “ejemplar”, mientras que, en el equipo médico y de fisioterapia, predominó el nivel “ejemplar”.

## INTRODUCTION

In the context of a patient’s health, it is extremely important that nurses and the entire multidisciplinary team have good clinical reasoning, a condition that can make the difference between life and death for many patients. In intensive care, patients are even more vulnerable, with more significant complications and difficulties, such as cardiopulmonary arrest (CPA)^(1)^.

CPA is often the terminal event following the progression and decompensation of a wide variety of pathophysiological events. With advances in healthcare and improvements in pre-hospital and in-hospital care provision, the occurrence of increasing rates of return of spontaneous circulation after CPA has been evidenced^(2)^. After cardiopulmonary resuscitation (CPR), the next challenge is to manage these patients appropriately, in order to not only prevent mortality but also preserve neurological and cognitive function^(3)^, making the need for clinical reasoning even more relevant^(4)^.

Clinical judgment is influenced by each experience that professionals have had throughout their career. According to Tanner, each professional’s actions develop throughout four phases: noticing; interpreting; responding; and reflecting^(5)^.

In the noticing phase, observation and recognition of what differentiates the case of physiology and how professionals will seek their information are assessed. In the interpreting phase, priority is given to understanding what was assessed in the previous phase. The responding phase reflects the format of actions and whether communication was clear and the intervention well planned. And in the reflecting phase, aspects of self-assessment and commitment to improvement based on the experience of each scenario are considered^(5)^.

Clinical judgment has been assessed using the Lasater Scale^(6)^, which is used to check whether reasoning is satisfactory. In this way, it is possible to assess and intervene in each professional for a significant change in their skills and, thus, verify in which area, considering the scale’s thinking, there is a need for improvement, especially when it comes to interprofessional education (IPE), a process in which two or more specialists learn together, learn from each other and learn about each other^(7)^.

Reports indicate that IPE is safe and effective for teaching basic skills and knowledge, and also improves communication among professional groups^(8)^. Therefore, IPE may ultimately lead to improved patient safety and outcomes^(9)^ by considering clinical reasoning and judgment. Therefore, better understanding the responses and relationships of IPE to improve healthcare professionals’ competencies may clarify gaps and better address uncertainties in patient care systematization in CPA with COVID-19.

## OBJECTIVES

To assess the effect of active teaching strategies on clinical judgment for CPA care of patients with COVID-19 in intra-hospital settings by an interprofessional team.

## METHODS

### Ethical aspects

This project was submitted to and approved by the Research Ethics Committee. Participants expressed voluntary acceptance to participate in the study by signing the Informed Consent Form (ICF), in accordance with Resolution 466 of December 12, 2012 of the Brazilian National Health Council.

### Study design, period and location

This is a study with quasi-experimental design, without comparison group. The Transparent Reporting of Nonrandomized Designs (TREND)^(10)^, through a checklist for reporting intervention assessment studies with non-randomized designs, made available by the EQUATOR network (https://www.equator-network.org/), was used in the planning and description of the present study.

The educational intervention was carried out in a room of the Adult Intensive Care Unit (ICU) of a tertiary teaching hospital in midwestern Brazil, consisting of a total of 19 beds, ten of which were General ICU beds and nine Coronary ICU beds, from December 2022 to January 2023.

### Population and inclusion and exclusion criteria

The population consisted of 106 healthcare professionals working in the ICU. The sample was non-probabilistic and intentional, consisting of 85 healthcare professionals (eight physicians, eight physiotherapists, 16 nurses, 46 nursing technicians and seven resident professionals, three from physiotherapy and four from medicine).

Healthcare professionals of both sexes, aged 18 or over, working in direct care for patients admitted to the ICU were included. Professionals on leave or vacation and those working only in administrative and/or management roles were excluded.

### Study protocol

#### Intervention

The educational intervention was carried out in two stages: (1) expository class on CPA aimed at patients with COVID-19; and (2) skills training based on IPE, using a medium-fidelity patient simulator (Resusci Anne-Laerdal^®^), on assistance in in-hospital CPA situations in critically ill patients with COVID-19 during the two-hour in-person period.

#### Instruments and scales

A demographic and professional identification questionnaire was used consisting of closed-ended questions regarding sex, profession, time passed since graduation, length working in the ICU, participation in a basic or advanced life support course, and a clinical judgment scale - Lasater Clinical Judgment Rubric (LCJR) -, indicated to assess the teaching-learning process performance, developed by Lasater and adapted to Brazilian culture as Lasater Clinical Judgment Rubric-Brazilian Version (LCJR-BV)^(11)^.

Clinical judgment was defined as a group of skills that comprises the synthesis of professionals’ knowledge and previous experience for responsible decision-making^(12)^.

In the professional environment, the LCJR allows assessing nurses’ performance in completing an educational activity and in self-assessment of strengths and weaknesses regarding competency in clinical judgment skills through reflective activity^(11)^. It presents 11 dimensions, which comprise the behaviors, verbalizations or actions that represent clinical judgment skills, based on four aspects of the clinical judgment model, designated as: noticing; interpreting; responding; and reflecting^(11,12)^.

The noticing phase was characterized by focused observation assessment, i.e., when deviations from expected patterns were recognized and information was sought. The interpreting phase corresponded to data prioritization and understanding. The responding phase represented the reflection of dimensions directed towards calm and confident action, clear communication, well-planned intervention/flexibility and technical skill. The reflecting phase represented aspects related to assessment/self-analysis and commitment to improvement^(11)^.

#### Data collection procedures

Phase 1. Professionals’ awareness was preceded by a meeting with the unit’s management team/leadership to present the study objectives and obtain authorization to begin data collection.

Phase 2. Forwarding of educational material on basic and advanced life support^(13)^ for the preparation and leveling of the team of professionals 30 days in advance of the educational intervention. In this phase, the schedule with the activities that constituted the study was made available.

Phase 3. After voluntary consent to participate in the study, professionals, before starting their shift, were taken to a reserved area of the ICU to complete a pre-training activity questionnaire for an estimated 10 minutes, under the supervision of the main researcher.

Phase 4. As an educational intervention, a dialogued lecture was given combined with skills training in the private intensive care environment itself to increase professionals’ adherence and reduce travel, which could have repercussions on patient care. The intervention lasted 60 minutes and was replicated for nine days to reach the majority of professionals.

Multimedia was used in the class to project and share actions and a video demonstrating the correct dressing maneuver during care of patients with COVID-19, in addition to topics such as in-hospital chain of survival, basic life support (BLS), advanced life support (ALS), types of CPA, shockable and non-shockable rhythms, and care in CPA in COVID-19.

For skills training, Resusci Anne-Laerdal^®^ mid-fidelity patient simulator was used, which allows CPR maneuvers, such as cardiac compression and airway ventilation^(14)^, bag-valve-mask device, and personal protective equipment. In skills training, maneuvers for opening and managing the airway, cardiac compression maneuvers, and CPR itself were demonstrated in accordance with the American Heart Association (AHA) (2020) guidelines^(13)^.

Phase 5. A clinical judgment scale was applied for approximately 15 minutes after training on CPA directed at patients with COVID-19, under the supervision of the main researcher, to avoid consultation and access to scientific sources on the web, which could mask the results.

#### Analysis of results, and statistics

Descriptive analysis was performed by calculating absolute and relative percentage frequencies. Continuous variables were described using mean, median, standard deviation, and interquartile range. The hypothesis of independence among categorical variables was tested using Pearson’s chi-square test. The hypothesis of adherence of continuous variables to a normal distribution was tested using the Shapiro-Wilk test; as this was not confirmed in any case, the hypothesis of equality of medians was tested between three or more independent groups using the Kruskal-Wallis test. All analyses were performed using R Core Team 2023 (Version 4.2.3), and the significance level adopted was 5%.

## RESULTS

Eighty-five healthcare professionals participated in the study, predominantly female (55; 64.7%). Nursing technicians (46; 54.1%) constituted more than half of the team, whereas nurses (16; 18.8%), physicians and physiotherapists, 9.4% each. We found that 8.2% of professionals worked as residents. Most professionals self-reported having graduated from a private institution (60%). *Lato sensu* graduate studies were declared by 52.9% of professionals, and *stricto sensu*, by 8.3%. Participation in BLS and ALS courses was reported, respectively, by professionals (82.8% vs. 34.4%) (Table 1).

**Table 1:** Characterization of healthcare professionals working in the Intensive Care Unit, Brasília, Federal District, Brazil, 2023

Variables	n	%
Sex		
Female	55	64.7
Male	30	35.3
Profession		
Nursing technician	46	54.1
Nurse	16	18.8
Physiotherapy	8	9.4
Physician	8	9.4
Resident	7	8.2
Technician with degree		
Yes	25	54.3
No	21	45.7
Training institution		
Public	34	40.0
Private	51	60.0
Level of education		
Vocational training	22	25.9
Undergraduate degree	11	12.9
Specialization lato sensu	45	52.9
Master’s degree stricto sensu	6	7.1
Doctoral degree stricto sensu	1	1.2
Time		
Time since graduation (years)	12,4	6.4
Length working in the Intensive Care Unit (months)	91,8	66.8
Background of participation in BLS course	53	82.8
History of participation in ALS course	22	34.4

* *n - absolute frequency; % - relative percentage frequency; BLS - basic life support; ALS - advanced life support.*

The first skill, noticing, of a clinical judgment scale showed a mean score of 9.4, with a standard deviation of 1.9, which indicates that most professionals obtained a high score and close to the mean. In the second skill assessed, interpreting, the mean score for this skill was 6.3, with a standard deviation of 1.3, which indicates that most professionals had a score below the mean. The third skill assessed was responding, with a mean score of 12.7 and a standard deviation of 2.6, indicating that most professionals had a score close to the mean. The reflecting skill showed a median of 6, with an interquartile range of 6 to 7, which suggests that the distribution of results was relatively symmetric.

The analysis of the overall measure of the clinical judgment scale showed the combination of the four skills assessed with an average score of 34.8, suggesting, in all domains, relatively similar results. When comparing the difference between professions, it was not possible to identify significant differences in any dimension or in the complete clinical judgment scale (Table 2).

**Table 2:** Assessment of professionals from different health areas in four domains, such as noticing, interpreting, responding and reflecting (clinical judgment scale), after intervention, Brasília, Federal District, Brazil, 2023

	Profession						
	Total	NT	NUR	PHY	PHYS	RES	*p* value
Noticing							
Mean (SD)	9.4 (1.9)	9.2 (1.8)	10 (1.8)	10.5 (1.2)	9.9 (1.9)	7.7 (2.4)	0.060
Median [IIQ]	9 [9-11]	9 [8-11]	10 [9-11.5]	10.5 [9.5-11.5]	10.5 [9-11]	9 [7-9]	
Interpreting							
Mean (SD)	6.3 (1.3)	6.2 (1.4)	6.4 (1.3)	6.5 (0.9)	7 (1.1)	5.4 (1.4)	0.343
Median [IIQ]	6 [6-7]	6 [6-8]	6 [6-7]	6 [6-7]	7 [6-8]	6 [4-6.5]	
Responding							
Mean (SD)	12.7 (2.6)	12.5 (2.6)	13 (2.5)	14.4 (1.8)	14 (1.9)	10.1 (3)	0.051
Median [IIQ]	12 [12-15]	12 [11-14]	12.5 [12-15.5]	15 [12.5-16]	14 [12-16]	12 [7.5-12.5]	
Reflecting							
Mean (SD)	6.4 (1.2)	6.3 (1.2)	6.7 (1.1)	6.4 (1.3)	6.5 (0.9)	6.1 (1.5)	0.896
Median [IIQ]	6 [6-7]	6 [6-8]	6.5 [6-8]	6 [6-7.5]	6.5 [6-7]	7 [5-7]	
Clinical judgment scale							
Mean (SD)	34.8 (6.3)	34.2 (6.3)	36.1 (6)	37.8 (4.3)	37.4 (5.3)	29.4 (7.8)	0.199
Median [IIQ]	34 [33-39]	33.5 [30-38]	36 [33-41]	37 [34-41.5]	38 [33-42]	34 [22.5-35.5]	

*NT - nursing technician; NUR - nurse; PHY - physiotherapist; PHYS - physician; RES - resident; SD - standard deviation; IIQ - interquartile range; Kruskal-Wallis test.*

In the clinical judgment scale results, categorized by performance skill levels “beginning”, “developing”, “proficiency” and “exemplary”, we identified that most participants were categorized as “proficiency”, accounting for 52.9% of the total. The “exemplary” category accounted for 31.8% of the total. Only 2.4% of participants were categorized as “beginning”, and 12.9%, as “developing”.

Nurses were equally divided between proficiency and exemplary (43.8%). Physicians and physiotherapists stood out in the “exemplary” category (50% in both cases). No participants were identified in the “beginning” category among nurses and physiotherapists and no participants in the “exemplary” category among resident professionals. Hence, p-value suggests that there are no statistically significant differences among the clinical judgment scale categories in relation to the different professions included in the study (Table 3).

**Table 3:** Categorization of professionals by performance skill levels, such as “beginning”, “developing”, “proficiency” and “exemplary”, post-intervention, Brasília, Federal District, Brazil, 2023

Clinical judgment scale	Profession						*p* value
	Total n (%)	NT	NUR	PHY	PHYS	RES	
Beginning	2 (2.4)	1 (2.2)	0 (0)	0 (0)	0 (0)	1(14.3)	0.284
Developing	11 (12.9)	7 (15.2)	2 (12.5)	0 (0)	0 (0)	2 (28.6)	
Proficiency	45 (52.9)	26 (56.5)	7 (43.8)	4 (50)	4 (50)	4 (57.1)	
Exemplary	27 (31.8)	12 (26.1)	7 (43.8)	4 (50)	4 (50)	0 (0)	

*NT - nursing technician; NUR - nurse; PHY - physiotherapist; PHYS - physician; RES - resident; n - absolute frequency; % - relative percentage frequency; Pearson’s chi-square test.*

## DISCUSSION

The results showed that active educational interventions favor clinical judgment improvement and better performance of professional skills, although no significant difference in skills was found among professional categories based on the clinical judgment scale. The “noticing” skill aimed at in-hospital CPA of patients with COVID-19 was found to be similar among nursing professionals (nurse and nursing technician), physiotherapists, and physicians. The “responding” skill in CPA situations showed divergence, since physiotherapists and physicians obtained a slightly better response compared to nurses. In the general context, the clinical judgment scale showed that all professional categories tended to have lower performance in the ability to interpret and reflect. Proficiency and exemplary professionals were predominant in the category of nurses, and the exemplary professional category was more frequent in the team of physiotherapists and physicians, which reveals, overall, the team’s professional competency, given prolonged time of training and work in the ICU and history of participation in BLS and ALS courses.

Clinical judgment is imperative for emergency responders caring for acutely ill patients. Without optimal clinical judgment in emergency situations, patients are at risk for medical errors and failed resuscitation^(15)^. In the ICU, consistent supervision, through monitoring devices, and timely intervention are crucial for managing the various clinical conditions among patients, with experience and training time being differential factors that contribute to faster and more assertive decision-making^(16)^, as evidenced by the professionals in the present study, who reported a long period of experience and academic training, which culminated in proficiency and exemplary professional performance of skills.

Recent study indicates that only 23% of new nursing graduates are competent with basic clinical judgment skills^(17)^. In the current investigation, nurses were equally distributed between proficiency and exemplary, but this result was still different from that presented by physiotherapists and physicians, who stood out mainly as “exemplary”, according to the clinical judgment scale^(18)^. Following this premise, one of the recommendations highlighted in the Carnegie Foundation report refers to the need to contextualize new knowledge in practice settings and include experiential learning environments in the curricula to better develop skills^(17,19)^. Thus, expository classes, followed by skills training, as an educational strategy, can be promising for achieving better consolidation and anchoring of scientific knowledge.

Competency-based education is increasingly encouraged in healthcare, as described by Giddens *et al*.^(20)^, in order to better prepare students. It has proven imperative for training programs to create deliberate learning experiences that are capable of qualifying graduates for an ever-changing healthcare scenario^(18)^, such as CPA care guidelines.

The *Conselho Nacional dos Conselhos Estaduais de Enfermagem* (NCSBN, Brazilian National Council of State Boards of Nursing) conducted a practical analysis that highlighted healthcare settings as increasingly complex and, therefore, the need for informed clinical judgment and decision-making skills has gained scientific evidence^(18)^.

Nurses work in teams with other healthcare professionals, which increasingly places value on IPE. IPE educators use a collaborative practice framework that outlines competencies for healthcare professionals working in teams^(21)^. Thus, a strategy such as expository classes combined with skills training has proven to be a valuable alternative for the growth and consolidation of knowledge of different professionals, as carried out in the present investigation.

The lack of professional experience and the inability to relate the theory studied to interpretation in clinical practice represent a major obstacle in the construction of professionals’ clinical reasoning, which highlights the need for more time to develop correlation and professional training, in addition to more attention and guidance from those who have already been in the area and employed for longer^(22)^, considering that skills such as interpreting and reflecting on situations experienced in practice in our study can be further improved through educational training strategies.

Dynamic training strategies, such as the activity carried out in the present study, entitled “lecture combined with skills training”, can favor the achievement of different levels of learning and encourage the learning of critical reasoning skills, decision-making, clinical interpretation, in addition to teamwork^(23)^.

Hence, balancing the different levels of knowledge to meet the requirements of today’s society must be encouraged to achieve an expectation of academic and professional autonomy and freedom. Therefore, the proposition of dynamic and interactive strategies, such as the educational intervention carried out, can promote greater interprofessional team interaction and collaboration^(24)^.

### Study limitations

The limitations of this study include the fact that it was developed at a single center, which limits the possibility of generalizing the results, as well as the small sample size. It does not have randomization and a control group.

### Contributions to health, nursing and public policies

Findings about nurses’ and healthcare professionals’ clinical judgment based on physiological signs of patients undergoing CPA may contribute to directing the development of collaborative strategies aimed at supporting the earlier identification of indicators of clinical worsening of patients and patient safety in a systematic manner. Educational intervention studies may be timely and crucial for improving clinical judgment and effective management of patients’ clinical conditions by a interprofessional team.

## CONCLUSIONS

An active strategy based on a dialogued lecture combined with skills training proved to be promising and had a positive impact on improving clinical judgment, even in an experienced interprofessional team. Thus, participatory educational actions, based on an active teaching method, developed, in most nurses, the “proficiency” and “exemplary” levels, whereas, in the medical and physiotherapy team, the “exemplary” level predominated in care for critically ill patients with COVID-19 in CPA.
