# Supplementary material for: Effect of active teaching strategies on interprofessional clinical judgment: a quasi-experiment
Source: Rev Bras Enferm. 2025 Mar 14;78(1):e20240148. doi: 10.1590/0034-7167-2024-0148 (PMC11913043; doi:10.1590/0034-7167-2024-0148)
Supplement: Translation

# Translation: Efeito de estratégias ativas de ensino no julgamento clínico interprofissional: quase-experimento

**DOI:** 10.1590/0034-7167-2024-0148pt

**Authors:** Susi Cristalino Pereira, Mikael Lucas Anjo Pires, Paulo Percio Mota Magro, Tayse Tâmara da Paixão Duarte, Marcia Cristina da Silva Magro

**Affiliations:** I Hospital Universitário de Brasília. Brasília, Distrito Federal, Brasil; II Universidade de Brasília. Brasília, Distrito Federal, Brasil; II Instituto Federal de Brasília. Brasília, Distrito Federal, Brasil

**Keywords:** Julgamento Clínico, Educação Interprofissional, Ensino, Unidade de Terapia Intensiva, Parada Cardíaca

## Abstract

**Objetivos:**

avaliar o efeito de estratégias ativas de ensino no julgamento clínico para atendimento da parada cardiopulmonar de pacientes com COVID-19 no cenário intra-hospitalar por uma equipe interprofissional.

**Métodos:**

estudo quase-experimental, sem grupo de comparação. Foram selecionados por amostragem não probabilística 85 profissionais. A intervenção educativa foi uma aula combinada ao treino de habilidades. O nível de significância foi de 5%.

**Resultados:**

a maioria dos profissionais foi categorizada como “proficiente” em escala de julgamento clínico (52,9%). A categoria “exemplar” representou 31,8% do total. Somente 2,4% dos participantes foram categorizados como “iniciante”, e 12,9%, “em desenvolvimento” pós-intervenção.

**Conclusões:**

estratégia ativa baseada em aula expositiva dialogada combinada ao treino de habilidades impactou positivamente o aperfeiçoamento do julgamento clínico. Assim, ações educativas participativas, a partir de método ativo de ensino, desenvolveram, na maioria dos enfermeiros, os níveis “proficiência” e “exemplar”, enquanto que, na equipe médica e de fisioterapia, predominou o nível “exemplar”.

## INTRODUÇÃO

No âmbito da saúde de um paciente, é extremamente importante que o enfermeiro e toda a equipe multiprofissional tenham um bom raciocínio clínico, condição que pode impactar a diferença entre a vida e a morte de muitos pacientes. Em cuidados intensivos, a vulnerabilidade dos pacientes é ainda maior, com complicações e dificuldades mais significativas, como a parada cardiopulmonar (PCP)^(1)^.

A PCP é frequentemente o evento terminal após a progressão e descompensação de uma ampla variedade de eventos fisiopatológicos. Com os avanços na área da saúde e melhorias na prestação de cuidados pré-hospitalares e intra-hospitalares, a ocorrência de taxas crescentes de retorno da circulação espontânea após a PCP tem sido evidenciada^(2)^. Após a reanimação cardiopulmonar (RCP), o próximo desafio é gerir esses pacientes de forma adequada, de modo a não só prevenir a mortalidade, mas também preservar a função neurológica e cognitiva^(3)^, sendo, portanto, a necessidade do raciocínio clínico ainda mais relevante^(4)^.

O julgamento clínico é influenciado por cada experiência que o profissional vivenciou ao longo de sua carreira. De acordo com Tanner, as ações de cada profissional se desenvolvem ao longo de quatro fases: reconhecimento (*noticing*); interpretação (*interpreting*); resposta (*responding*); e reflexão (*reflecting*)^(5)^.

Na fase de reconhecimento, avaliam-se a observação e o reconhecimento daquilo que diferencia o caso da fisiologia e como o profissional buscará suas informações. Na fase de interpretação, prioriza-se a compreensão sobre o que foi avaliado na fase anterior. A fase de resposta reflete o formato das atuações e se a comunicação foi clara e a intervenção bem planejada. E na fase de reflexão, são considerados aspectos da autoavaliação e comprometimento com a melhoria a partir da vivência de cada cenário^(5)^.

O julgamento clínico tem sido avaliado a partir da Escala de Lasater^(6)^, utilizada para verificar se o raciocínio está sendo satisfatório. Dessa forma, é possível avaliar e intervir em cada profissional para uma mudança significativa de suas habilidades e, assim, verificar em qual âmbito, diante do pensamento da escala, está precisando melhorar, especialmente quando se trata da educação interprofissional (EIP), processo no qual dois ou mais especialistas aprendem juntos, aprendem uns com os outros e aprendem uns sobre os outros^(7)^.

Relatórios indicam que a EIP é segura e eficaz para o ensino de habilidades e conhecimentos básicos, e também melhora a comunicação entre grupos profissionais^(8)^. Logo, a EIP pode, em última análise, levar a uma melhor segurança e resultados do paciente^(9)^, considerando o raciocínio e julgamento clínicos. Então, compreender melhor as respostas e relações da EIP para aperfeiçoamento das competências de profissionais de saúde pode esclarecer lacunas e direcionar melhor as incertezas na sistematização da assistência ao paciente em PCP com COVID-19.

## OBJETIVOS

Avaliar o efeito de estratégias ativas de ensino no julgamento clínico para atendimento da PCP de pacientes com COVID-19 no cenário intra-hospitalar por uma equipe interprofissional.

## MÉTODOS

### Aspectos éticos

Este projeto foi submetido e aprovado pelo Comitê de Ética em Pesquisa. Os participantes expressaram aceite voluntário para participar do estudo por meio de assinatura do Termo de Consentimento Livre e Esclarecido (TCLE), conforme Resolução nº 466, de 12 de dezembro de 2012, do Conselho Nacional de Saúde.

### Desenho, período e local do estudo

Estudo com delineamento quase-experimental, sem grupo de comparação. A instrução *Transparent Reporting of Nonrandomized Designs* (TREND)^(10)^, através de um *checklist* para o relato de estudos de avaliação de intervenções com desenhos não randomizados, disponibilizada pela rede EQUATOR (*https://www.equator-network.org/*), foi utilizada no planejamento e descrição do presente estudo.

A intervenção educativa foi realizada em um quarto da Unidade de Terapia Intensiva (UTI) Adulto de um hospital terciário de ensino da região Centro-Oeste do Brasil, constituída de um total de 19 leitos, sendo dez leitos de UTI Geral e nove leitos de UTI Coronariana, durante o período de dezembro de 2022 a janeiro de 2023.

### População e critérios de inclusão e exclusão

A população foi constituída de 106 profissionais de saúde atuantes na UTI. A amostra foi não probabilística e intencional, constituída de 85 profissionais de saúde (oito médicos, oito fisioterapeutas, 16 enfermeiros, 46 técnicos de enfermagem e sete profissionais residentes, sendo três da fisioterapia e quatro da medicina).

Foram incluídos profissionais de saúde de ambos os sexos, com idade igual ou superior a 18 anos, atuantes na assistência direta aos pacientes internados na UTI. Foram excluídos profissionais em período de licença ou férias e com exercício profissional somente em funções administrativas e/ou de gerência.

### Protocolo do estudo

#### Intervenção

A intervenção educativa foi realizada em duas etapas: (1) aula expositiva sobre o tema PCP direcionada ao paciente com COVID-19; e (2) treino de habilidades baseado na EIP, com uso de um simulador de paciente de média fidelidade (*Resusci Anne-Laerdal* ^®^), sobre assistência em situação de PCP intra-hospitalar em pacientes críticos com COVID-19 durante o período de duas horas presenciais.

#### Instrumentos e escalas

Foram utilizados questionário de identificação demográfica e profissional, constituído de questões fechadas referentes ao sexo, profissão, tempo de formação, tempo de experiência em UTI, participação em curso de suporte básico ou avançado de vida, e uma escala de julgamento clínico - *Lasater Clinical Judgment Rubric* (LCJR) -, indicada para avaliar o desempenho do processo de ensino-aprendizagem, desenvolvida por Lasater e adaptada à cultura brasileira como *Lasater Clinical Judgment Rubric-Brazilian Version* (LCJR-BV)^(11)^.

O julgamento clínico foi definido como grupo de habilidades que compreende a síntese do conhecimento e da experiência pregressa do profissional para a tomada de decisão responsável^(12)^.

No ambiente profissional, a LCJR permite avaliar o desempenho dos enfermeiros na finalização de uma atividade educacional e na autoavaliação de pontos fortes e fracos quanto à competência na habilidade de julgamento clínico através da atividade reflexiva^(11)^. Apresenta 11 dimensões, que compreendem os comportamentos, verbalizações ou ações que representam habilidades de julgamento clínico, fundamentada em quatro aspectos do modelo de julgamento clínico, designado como: reconhecimento (*noticing*); interpretação (*interpreting*); resposta (*responding*); e reflexão (*reflecting*)^(11,12)^.

A fase de reconhecimento se caracterizou pela avaliação da observação focada, ou seja, quando ocorreram o reconhecimento de desvios dos padrões esperados e a busca por informações. A fase de interpretação correspondeu à priorização e compreensão dos dados. A fase de resposta representou a reflexão das dimensões direcionadas para atuação calma e confiante, comunicação clara, intervenção bem planejada/flexibilidade e habilidade técnica. A fase de reflexão representou os aspectos relacionados à avaliação/autoanálise e o comprometimento com o aperfeiçoamento^(11)^.

#### Procedimentos de coleta de dados

Fase 1. Sensibilização dos profissionais foi antecedida pela reunião com a chefia/equipe de gestão da unidade para apresentação dos objetivos do estudo e obtenção da autorização para o início da coleta de dados.

Fase 2. Encaminhamento de material didático sobre suporte básico e avançado de vida^(13)^, para preparação e nivelamento da equipe de profissionais, com antecedência de 30 dias da intervenção educativa. Nessa fase, foi disponibilizado o cronograma com a programação das atividades que constituíram o estudo.

Fase 3. Após consentimento voluntário para participação do estudo, os profissionais, antes de iniciar o plantão, foram conduzidos ao ambiente reservado da UTI para preenchimento do questionário pré-atividade de capacitação durante estimadamente 10 minutos, sob supervisão da pesquisadora principal.

Fase 4. Como intervenção educativa, ministrou-se aula expositiva dialogada combinada a treino de habilidades no próprio ambiente privativo de terapia intensiva para maior aderência dos profissionais e redução de deslocamentos com possíveis repercussões à assistência aos pacientes. A intervenção teve duração de 60 minutos, e foi replicada por nove dias para alcançar a maioria dos profissionais.

Foram usados para aula o multimídia para projeção e compartilhamento de ações e um vídeo demonstrativo da manobra de paramentação correta durante cuidado do paciente com COVID-19, além de ter sido abordados os tópicos sobre cadeia de sobrevivência intra-hospitalar, suporte básico de vida (SBV), suporte avançado de vida (SAV), tipos de PCP, ritmos chocáveis e não chocáveis, cuidados na PCP na COVID-19.

Para o treino de habilidades, foi usado simulador de paciente de média fidelidade modelo *Resusci Anne-Laerdal* ^®^, que permite manobras de RCP, como compressão cardíaca e ventilação de vias aéreas^(14)^, dispositivo bolsa-válvula-máscara, equipamentos de proteção individual. No treinamento de habilidades, foram demonstradas manobras para abertura e manejo da via aérea, manobras de compressão cardíaca e a própria RCP obedecendo às diretrizes da *American Heart Association* (AHA) (2020)^(13)^.

Fase 5. Aplicação da escala de julgamento clínico ocorreu durante aproximadamente 15 minutos pós-capacitação sobre PCP direcionada ao paciente com COVID-19, sob a supervisão da pesquisadora principal, para evitar consulta e acesso a *web* de fontes científicas, o que pudesse mascarar os resultados.

#### Análise dos resultados e estatística

Realizou-se análise descritiva por meio de cálculo de frequência absoluta e relativa percentual. As variáveis contínuas foram descritas por meio de média, mediana, desvio padrão e intervalo interquartil. A hipótese de independência entre variáveis categóricas foi testada por meio do teste qui-quadrado de Pearson. A hipótese de aderência das variáveis contínuas à distribuição normal foi testada pelo teste de Shapiro-Wilk; como esta não foi confirmada em nenhum caso, a hipótese de igualdade de medianas foi testada entre três ou mais grupos independentes por meio do teste de Kruskal-Wallis. Todas as análises foram realizadas no *software* R Core Team 2023 (Versão 4.2.3), e o nível de significância adotado foi de 5%.

## RESULTADOS

Participaram do estudo 85 profissionais de saúde, predominantemente do sexo feminino (55; 64,7%). Os técnicos de enfermagem (46; 54,1%) constituíram mais da metade da equipe, enquanto que os enfermeiros (16; 18,8%), médicos e fisioterapeutas, 9,4% cada. Constatamos que 8,2% dos profissionais ocupavam a função de residente. A maioria dos profissionais autorreferiu formação em instituição privada (60%). A pós-graduação *lato sensu* foi declarada por 52,9% dos profissionais, e *stricto sensu*, por 8,3%. A participação em curso de SBV e de SAV foi informada, respectivamente, pelos profissionais (82,8% vs. 34,4%) (Tabela 1).

**Tabela 1:** Caracterização dos profissionais de saúde atuantes na Unidade de Terapia Intensiva, Brasília, Distrito Federal, Brasil, 2023

Variáveis	n	%
Sexo		
Feminino	55	64,7
Masculino	30	35,3
Profissão		
Técnico de enfermagem	46	54,1
Enfermeiro	16	18,8
Fisioterapia	8	9,4
Médico	8	9,4
Residente	7	8,2
Técnico com graduação		
Sim	25	54,3
Não	21	45,7
Formação de instituição		
Pública	34	40,0
Privada	51	60,0
Nível de escolaridade		
Técnico	22	25,9
Graduação	11	12,9
Especialização lato sensu	45	52,9
Mestrado stricto sensu	6	7,1
Doutorado stricto sensu	1	1,2
Tempo		
Tempo de formado (anos)	12,4	6,4
Tempo de Unidade de Terapia Intensiva (meses)	91,8	66,8
Antecedente de participação em curso de SBV	53	82,8
Antecedente de participação em curso de SAV	22	34,4

* *n - frequência absoluta; % - frequência relativa percentual; SBV - suporte básico de vida; SAV - suporte avançado de vida.*

A primeira habilidade sobre reconhecimento da escala de julgamento clínico mostrou média de pontuação de 9,4, com um desvio padrão de 1,9, o que indica que a maioria dos profissionais obteve uma pontuação alta e próxima à média. Na segunda habilidade avaliada, interpretação, a média de pontuação para essa habilidade foi de 6,3, com um desvio padrão de 1,3, o que indica que a maioria dos profissionais teve uma pontuação abaixo da média. A terceira habilidade avaliada foi a resposta, com a média de pontuação de 12,7 e desvio padrão de 2,6, indicando uma maioria de profissionais com pontuação próxima à média. A habilidade de reflexão evidenciou uma mediana de 6, com um intervalo interquartil de 6 a 7, o que sugere que a distribuição dos resultados foi relativamente simétrica.

A análise da medida geral da escala de julgamento clínico mostrou a combinação das quatro habilidades avaliadas com média de pontuação de 34,8, sugerindo, em todos os domínios, resultados relativamente simétricos. Ao comparar a diferença entre as profissões, não foi possível identificar diferenças significativas em nenhuma dimensão ou na escala completa de julgamento clínico (Tabela 2).

**Tabela 2:** Avaliação dos profissionais de diferentes áreas da saúde em quatro domínios, como reconhecimento, interpretação, resposta e reflexão (escala de julgamento clínico), após intervenção, Brasília, Distrito Federal, Brasil, 2023

	Profissão						
	Total	TE	EN	FIS	MÉD	RES	Valor de *p*
Reconhecimento							
Média (DP)	9,4 (1,9)	9,2 (1,8)	10 (1,8)	10,5 (1,2)	9,9 (1,9)	7,7 (2,4)	0,060
Mediana [IIQ]	9 [9-11]	9 [8-11]	10 [9-11,5]	10,5 [9,5-11,5]	10,5 [9-11]	9 [7-9]	
Interpretação							
Média (DP)	6,3 (1,3)	6,2 (1,4)	6,4 (1,3)	6,5 (0,9)	7 (1,1)	5,4 (1,4)	0,343
Mediana [IIQ]	6 [6-7]	6 [6-8]	6 [6-7]	6 [6-7]	7 [6-8]	6 [4-6,5]	
Resposta							
Média (DP)	12,7 (2,6)	12,5 (2,6)	13 (2,5)	14,4 (1,8)	14 (1,9)	10,1 (3)	0,051
Mediana [IIQ]	12 [12-15]	12 [11-14]	12,5 [12-15,5]	15 [12,5-16]	14 [12-16]	12 [7,5-12,5]	
Reflexão							
Média (DP)	6,4 (1,2)	6,3 (1,2)	6,7 (1,1)	6,4 (1,3)	6,5 (0,9)	6,1 (1,5)	0,896
Mediana [IIQ]	6 [6-7]	6 [6-8]	6,5 [6-8]	6 [6-7,5]	6,5 [6-7]	7 [5-7]	
Escala de julgamento clínico							
Média (DP)	34,8 (6,3)	34,2 (6,3)	36,1 (6)	37,8 (4,3)	37,4 (5,3)	29,4 (7,8)	0,199
Mediana [IIQ]	34 [33-39]	33,5 [30-38]	36 [33-41]	37 [34-41,5]	38 [33-42]	34 [22,5-35,5]	

*TE - técnico de enfermagem; EN - enfermeiro; FIS - fisioterapeuta; MÉD - médico; RES - residente; DP - desvio padrão; IIQ - intervalor interquartil; teste de Kruskal-Wallis.*

Nos resultados da escala de julgamento clínico, categorizados por níveis de habilidade de desempenho “inicial”, “em desenvolvimento”, “proficiente” e “exemplar”, identificamos que a maioria dos participantes foi categorizada como “proficiente”, representando 52,9% do total. A categoria “exemplar” representou 31,8% do total. Somente 2,4% dos participantes foram categorizados como “inicial”, e 12,9%, como “em desenvolvimento”.

Os enfermeiros mostraram-se em maioria igualmente subdivididos entre proficientes e exemplares (43,8%). Os médicos e fisioterapeutas se destacaram na categoria “exemplar” (50% em ambos os casos). Não foram identificados participantes na categoria “inicial” entre enfermeiros e fisioterapeutas e nenhum participante da categoria “exemplar” entre os profissionais residentes. O valor de p sugere que não há diferenças estatisticamente significativas entre as categorias da escala de julgamento clínico em relação às diferentes profissões incluídas no estudo (Tabela 3).

**Tabela 3:** Categorização dos profissionais por níveis de habilidade de desempenho, como “inicial”, “em desenvolvimento”, “proficiente” e “exemplar”, pós-intervenção, Brasília, Distrito Federal, Brasil, 2023

Escala de julgamento clínico	Profissão						Valor de *p*
	Total n (%)	TE	EN	FIS	MÉD	RES	
Inicial	2 (2,4)	1 (2,2)	0 (0)	0 (0)	0 (0)	1(14,3)	0,284
Em desenvolvimento	11 (12,9)	7 (15,2)	2 (12,5)	0 (0)	0 (0)	2 (28,6)	
Proficiente	45 (52,9)	26 (56,5)	7 (43,8)	4 (50)	4 (50)	4 (57,1)	
Exemplar	27 (31,8)	12 (26,1)	7 (43,8)	4 (50)	4 (50)	0 (0)	

*TE - técnico de enfermagem; EN - enfermeiro; FIS - fisioterapeuta; MÉD - médico; RES - residente; n - frequência absoluta; % - frequência relativa percentual; teste qui-quadrado de Pearson.*

## DISCUSSÃO

Os resultados mostraram que intervenções educativas ativas favorecem o aperfeiçoamento do julgamento clínico e um melhor desempenho das competências profissionais, ainda que não tenha sido verificada diferença significativa das habilidades entre as categorias profissionais a partir da escala de julgamento clínico. A habilidade de “reconhecimento” direcionada à PCP intra-hospitalar de pacientes com COVID-19 mostrou-se similar entre profissionais de enfermagem (enfermeiro e técnico de enfermagem), fisioterapeuta e médico. A habilidade “resposta” em situação de PCP mostrou divergência, visto que o fisioterapeuta e o médico obtiveram uma resposta pouco melhor em relação ao enfermeiro. No contexto geral, a escala de julgamento clínico mostrou que todas as categorias profissionais apresentaram tendência a um menor desempenho na capacidade de interpretação e reflexão. Foram predominantes na categoria de enfermeiros profissionais proficientes e exemplares, e na equipe de fisioterapeutas e médicos, foi mais frequente a categoria de profissional exemplar, o que revela, de forma global, competência profissional da equipe, haja vista o tempo de formação e atuação na UTI prolongados e histórico de participação em cursos de SBV e SAV.

O julgamento clínico é imperativo para o profissional de emergência que cuida de pacientes com doenças agudas. Sem um julgamento clínico ideal em situações de emergência, os pacientes correm o risco de erros médicos e de falha na reanimação^(15)^. Na UTI, a supervisão consistente, por meio de dispositivos de monitoramento, e a intervenção oportuna são cruciais para o gerenciamento das diversas condições clínicas entre os pacientes, sendo a experiência e o tempo de formação diferenciais que contribuem para tomada de decisão mais rápida e assertiva^(16)^, como evidenciado pelos profissionais do presente estudo, que informaram um grande período de experiência e de formação acadêmica, o que culminou em um desempenho profissional proficiente e exemplar das competências.

Estudo recente indica que apenas 23% dos novos graduados em enfermagem são competentes, com habilidades básicas de julgamento clínico^(17)^. Na atual investigação, os enfermeiros se mostraram distribuídos igualmente entre proficientes e exemplares, mas, ainda assim, esse resultado foi diferente do apresentado pelos profissionais fisioterapeutas e médicos, que se destacaram principalmente como “exemplares”, conforme a escala de julgamento clínico^(18)^. Seguindo essa premissa, uma das recomendações destacada no relatório da Fundação Carnegie se refere à necessidade de contextualização dos novos conhecimentos no cenário da prática e inclusão de ambientes de aprendizagem experiencial nos currículos para melhor desenvolvimento das competências^(17,19)^. Dessa forma, aulas expositivas, seguidas de treino de habilidades, como estratégia educativa, podem ser promissoras para alcance de melhor consolidação e ancoragem do conhecimento científico.

A educação baseada em competências é cada vez mais incentivada na área da saúde, conforme descrito por Giddens *et al*.^(20)^, a fim de preparar melhor os estudantes. Tem se mostrado imperativa para que os programas de capacitação criem experiências de aprendizagem deliberadas e capazes de qualificar os graduados para um cenário em constante mudança na área da saúde^(18)^, a exemplo das diretrizes de atendimento de PCP.

O Conselho Nacional dos Conselhos Estaduais de Enfermagem (NCSBN) realizou uma análise prática que destacou o ambiente de saúde como cada vez mais complexo e, portanto, a necessidade de um fundamentado julgamento clínico e competências de tomada de decisão tem ganhado evidência científica^(18)^.

Os enfermeiros trabalham em equipe com outras profissões da área da saúde, o que cada vez mais valoriza a EIP. Os educadores de EIP utilizam uma estrutura de prática colaborativa que descreve competências para profissionais de saúde que trabalham em equipe^(21)^. Dessa forma, estratégia como aula expositiva combinada com treino de habilidades tem se mostrado alternativa valiosa para o crescimento e consolidação do conhecimento de diferentes profissionais, como o realizado na presente investigação.

A falta de experiência profissional e a incapacidade de relacionar a teoria estudada à interpretação na prática clínica representam um grande obstáculo na construção do raciocínio clínico do profissional, o que ressalta a necessidade de mais tempo para desenvolvimento da correlação e da capacitação profissional, além de mais atenção e orientação daqueles que já estão na área e empregados há mais tempo^(22)^, considerando que habilidades como interpretação e reflexão sobre as situações vivenciadas na prática em nosso estudo podem ser ainda melhoradas a partir de estratégias educativas de capacitação.

Estratégias dinâmicas de capacitação, como a atividade realizada no presente estudo, intitulada “aula expositiva combinada ao treino de habilidades”, pode favorecer o alcance de diferentes níveis de aprendizado e estimular a aprendizagem de competências de raciocínio crítico, tomada de decisão, interpretação clínica, além de trabalho em equipe^(23)^.

Nessa direção, equilibrar os diferentes níveis de conhecimento para atender aos requisitos da sociedade atual deve ser estimulado para alcançar uma expectativa de autonomia e liberdade acadêmica e profissional, então, a proposição de estratégias dinâmicas e interativas, como a intervenção educativa realizada, pode promover maior interação e colaboração da equipe interprofissional^(24)^.

### Limitações do estudo

As limitações deste estudo percorrem o fato de ter sido desenvolvido em um único centro, o que limita a possibilidade de generalização dos resultados, assim como o pequeno tamanho amostral. Não possui randomização e grupo controle.

### Contribuições para as áreas da saúde, enfermagem e políticas públicas

Descobertas sobre o julgamento clínico de enfermeiros e profissionais de saúde com base em sinais fisiológicos dos pacientes em PCP podem contribuir para direcionar o desenvolvimento de estratégias colaborativas visando subsidiar a identificação mais precoce de indicadores da piora clínica dos pacientes e segurança do paciente de forma sistemática. Estudos de intervenção educativa podem ser oportunos e cruciais para o aperfeiçoamento do julgamento clínico e gerenciamento efetivo de condições clínicas dos pacientes pela equipe interprofissional.

## CONCLUSÕES

Estratégia ativa baseada em aula expositiva dialogada combinada ao treino de habilidades mostrou-se promissora e impactou positivamente o aperfeiçoamento do julgamento clínico, mesmo em uma equipe interprofissional com experiência. Assim, ações educativas participativas, a partir de método ativo de ensino, desenvolveram, na maioria dos enfermeiros, os níveis “proficiência” e “exemplar”, enquanto que, na equipe médica e de fisioterapia, predominou o nível “exemplar” no atendimento do paciente crítico com COVID-19 em PCP.
